# The Beneficial Effect of HES on Vascular Permeability and Its Relationship With Endothelial Glycocalyx and Intercellular Junction After Hemorrhagic Shock

**DOI:** 10.3389/fphar.2020.00597

**Published:** 2020-05-08

**Authors:** Hongliang Zhao, Yu Zhu, Jie Zhang, Yue Wu, Xinming Xiang, Zisen Zhang, Tao Li, Liangming Liu

**Affiliations:** State Key Laboratory of Trauma, Burns and Combined Injury, Shock and Transfusion Department, Research Institute of Surgery, Daping Hospital, Army Medical University, Chongqing, China

**Keywords:** hemorrhagic shock, vascular permeability, endothelial glycocalyx, hydroxyethyl starch, intercellular junction proteins

## Abstract

**Background:**

Vascular leakage is a common complication of hemorrhagic shock. Endothelial glycocalyx plays a crucial role in the protection of vascular endothelial barrier function. Hydroxyethyl starch (HES) is a commonly used resuscitation fluid for hemorrhagic shock. However, whether the protective effect of HES on vascular permeability after hemorrhagic shock is associated with the endothelial glycocalyx is unclear.

**Methods:**

Using hemorrhagic shock rat model and hypoxia treated vascular endothelial cells (VECs), effects of HES (130/0.4) on pulmonary vascular permeability and the relationship to endothelial glycocalyx were observed.

**Results:**

Pulmonary vascular permeability was significantly increased after hemorrhagic shock, as evidenced by the increased permeability of pulmonary vessels to albumin-fluorescein isothiocyanate conjugate (FITC-BSA) and Evans blue, the decreased transendothelial electrical resistance of VECs and the increased transmittance of FITC-BSA. The structure of the endothelial glycocalyx was destroyed, showing a decrease in thickness. The expression of heparan sulfate, hyaluronic acid, and chondroitin sulfate, the components of the endothelial glycocalyx, was significantly decreased. HES (130/0.4) significantly improved the vascular barrier function, recovered the thickness and the expression of components of the endothelial glycocalyx by down-regulating the expression of heparinase, hyaluronidase, and neuraminidase, and meanwhile increased the expression of intercellular junction proteins ZO-1, occludin, and VE-cadherin. Degradation of endothelial glycocalyx with degrading enzyme (heparinase, hyaluronidase, and neuraminidase) abolished the beneficial effect of HES on vascular permeability, but had no significant effect on the recovery of the expression of endothelial intercellular junction proteins induced by HES (130/0.4). HES (130/0.4) decreased the expression of cleaved-caspase-3 induced by hemorrhagic shock.

**Conclusions:**

HES (130/0.4) has protective effect on vascular barrier function after hemorrgic shock.The mechanism is mainly related to the protective effect of HES on endothelial glycocalyx and intercellular junction proteins. The protective effect of HES on endothelial glycocalyx was associated with the down-regulated expression of heparinase, hyaluronidase, and neuraminidase. HES (130/0.4) had an anti-apoptotic effect in hemorrhagic shock.

## Introduction

Hemorrhagic shock is an important cause of early traumatic mortality in both civilian and military populations (Bellamy, 1984; Cannon, 2018). Hemorrhagic shock has an incidence of 10–15% in war trauma, and accounts for 50% of war wound mortality (Bellamy, 1984; Liu et al., 2013). Fluid resuscitation is an essential measurement for the primary treatment of patients with hemorrhagic shock and the key to the survival of patients with traumatic shock. Fluid resuscitation can maintain hemodynamics and improve tissue perfusion (Bougle et al., 2013; Rossaint et al., 2016; Komori et al., 2019). The commonly used resuscitation fluids include crystalloid and colloid fluids (Bougle et al., 2013; Trieu et al., 2018). Hydroxyethyl starch (HES) is a commonly used colloid fluid. Many studies have demonstrated that HES may effectively maintain osmotic pressure, restore microcirculation, maintain hemodynamics, and tissue perfusion (Dubin et al., 2010; Finfer et al., 2010; Myburgh et al., 2012; Feldheiser et al., 2013; Wu et al., 2015). However, large volume of HES has some side effects such as coagulation dysfunction and renal dysfunction (Haase et al., 2013; Serpa et al., 2014).

Vascular leakage, a common complication of hemorrhagic shock and fluid resuscitation, can increase the colloid osmotic pressure in the interstitial space, cause interstitial edema, decrease the effective circulating blood volume, and ultimately aggravate the occurrence of multiple organ dysfunction (Duan et al., 2017). In clinical practice, decreasing the occurrence of vascular leakage in fluid resuscitation therapy is an important treatment for preventing organ dysfunction (Koning et al., 2018). HES, as a common clinical resuscitation fluid, has controversial effect on vascular leakage. Some researchers think that HES can reduce the vascular leakage after hemorrhagic shock (Balkamou et al., 2010), but others think that HES can aggravate the vascular leakage (Wong et al., 2016; Wong et al., 2019). Therefore, whether HES (130/0.4) is beneficial to vascular leakage in hemorrhagic shock, and its underlying mechanisms, need further investigation and confirmation.

Endothelial glycocalyx, a dynamic reticular structure that covers the endothelial surface of the vascular lumen, plays a crucial role in maintaining the vascular barrier function (Lennon and Singleton, 2011; Alphonsus and Rodseth, 2014; Martin et al., 2016; Pillinger and Kam, 2017). It consists of multiple components, including proteoglycans and glycosaminoglycans (GAGs), which are made of heparan sulfate (HS), hyaluronic acid (HA), and chondroitin sulfate (CS) (Alphonsus and Rodseth, 2014; Chelazzi et al., 2015). Pathological stimulation, such as hemorrhagic shock and sepsis, may destroy the structure and function of the endothelial glycocalyx, thereby increasing vascular permeability (Nordling et al., 2015; Han et al., 2016; Huang et al., 2016). Whether the protective effect of HES (130/0.4) on vascular leakage in hemorrhagic shock is associated with endothelial glycocalyx, and its specific mechanism, remain unclear.

In the present study, using rat hemorrhagic shock model and hypoxia-stimulated vascular endothelial cells (VECs), we investigated the protective effect of HES (130/0.4) on pulmonary vascular permeability after hemorrhagic shock and its relationship and mechanism to endothelial glycocalyx and intercellular junction proteins.

## Materials and Methods

### Ethical Approval of the Study Protocol

This study was approved by the Laboratory Animal Welfare and Ethics Committee of the Army Medical University, and conformed to the Guide for the Care and Use of Laboratory Animals, published by the US National Institutes of Health (NIH Publication, 8th edition, 2011). All experiments conformed to the guidelines for ethical use of animals, and all effort was made to minimize animal suffering and to limit the number of animals used.

### Establishment of Hemorrhagic Shock Model

**Figure 1:**
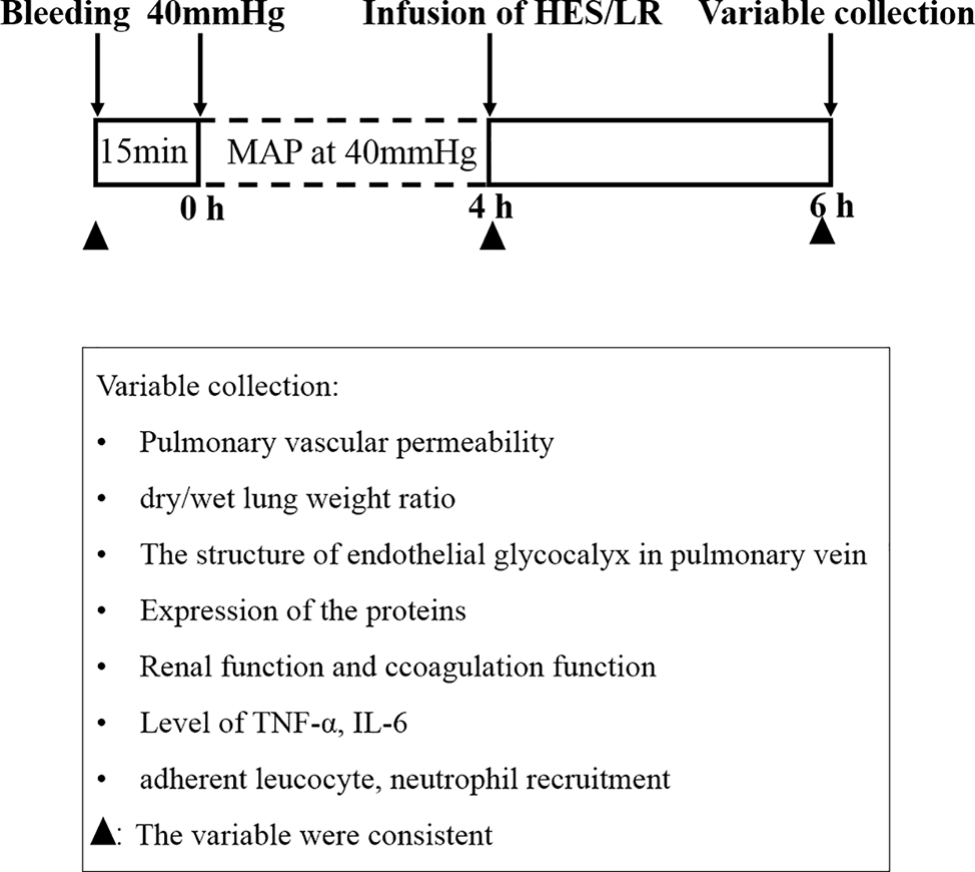
Experimental protocol (schematic).

Two hundred and eighty-eight female and male Sprague Dawley (SD) rats (half females and half males), weighing 200 to 220 g, were fasted for 12 h, but given water ad libitum and provide energy substances such as glucose in water before the experiment. The hemorrhagic shock model was prepared as previously described (Lei et al., 2015; Lei et al., 2019). Animals were anesthetized with 3% sodium pentobarbital (45 mg/kg i.p., Sigma, USA), and the right femoral artery and femoral vein of each animal were isolated and cannulated with polyethylene tubing (0.9×0.5 mm, microtube extrusions, Australia) for bleeding, monitoring the mean arterial pressure (MAP) and infusion. Blood was withdrawn through the right femoral artery catheter until the MAP decreased to 40 mmHg and maintained at this level for 4 h by blood withdrawal and a replacement as needed (**Figure 1**).

### Preparation of HES

HES was purchased from Voluven™, Fresenius Kabi, Bad Homburg, Germany. The main physicochemical characteristics of HES 130/0.4 are as follows: average mean molecular weight = 130 kDa; molar substitution = 0.4; C2/C6 ratio = 9, and concentration = 6%.

### Fluid Resuscitation of Hemorrhagic Shock

After the establishment of hemorrhagic shock model, rats received Ringer's lactate (LR) or HES (130/0.4) resuscitation. Rats in shock control group received LR two times the volume of blood loss at a speed of 20 ml/h, while rats in HES group received a mixture of HES and LR (HES: LR = 1:2) two times the volume of blood loss also at a speed of 20 ml/h. (**Supplementary Table 1**)

### Cell Culture and Hypoxia Treatment

VECs were isolated from pulmonary veins of SD rats and cultured in Dulbecco's modified Eagle's medium (DMEM)-F12 supplemented with 6% fetal bovine serum (Hyclone, Logan, UT, USA) and 1% penicillin-streptomycin (Hyclone, Logan, UT, USA). VECs were incubated at 37 °C in 5% CO_2_/95% O_2_. VECs were cultured in serum-free low-glucose DMEM and were transferred into a hypoxia culture compartment (MIC-101, Billups-Rothenberg, Del Mar, CA) equilibrated with 95% N_2_ and 5% CO_2_, in which the estimated oxygen concentration was less than 0.2% (Lei et al., 2019). Under hypoxic conditions, VECs were incubated for 4 h and then used for the following experiments.

### Measurement of Pulmonary Vascular Permeability in Rats With FITC-BSA/Evans Blue

Rats were anesthetized and fixed, and albumin-fluorescein isothiocyanate conjugate (FITC-BSA, 1.8 mg/ml, 9 mg/kg) or Evans blue (20 mg/ml, 60 mg/kg) was injected through the jugular vein. Measurement of pulmonary vascular permeability was performed as previously described (Zhang et al., 2015): the enterocoelia was opened along the linea alba abdominis after 2 h of FITC-BSA administration (or 30 min after Evans blue administration), the inferior vena cava was ligated, the abdominal aorta was cut, and phosphate buffer solution (PBS, 0.2 mol/L) was slowly perfused through the jugular vein until there were no hemocytes in the effluent (approximately 50 ml). The rats were then killed, and the complete lung was collected. The upper lobe of the left lung was dried and weighed, and PBS (PBS volume [ml] = 0.07 ml/g tissue) was added and homogenized in an ice-bath. The homogenate was transferred to a cryogenic centrifuge and centrifuged (8,000 g, 10 min, 4°C), and the supernatant was then centrifuged again (16,000 g, 10 min, 4°C). The optical density (OD) of the supernatant was determined with a spectrophotometer (excitation wavelength: 494 nm [FITC-BSA] or 562 nm [Evans blue]). In addition, the protein concentration in the supernatant was detected with a Pierce™ BCA protein assay kit (Thermo Scientific) according to the manufacturer's instructions (excitation wavelength: 562 nm) (Adilakshmi and Laine, 2002). Finally, the ratio of the OD value to the protein concentration in the supernatant was considered the pulmonary vascular permeability. Right lung tissue was embedded in Optimal Cutting Temperature compound, and frozen sections (10–15 μm thickness) were generated. The infiltration of FITC-BSA in the lung was observed on the basis of immunofluorescence under a laser confocal microscope (Leica Microsystems, Germany). The mean optical density was considered to reflect the infiltration of FITC-BSA in the lung. In the Evans blue group, water on the lung tissue surface was absorbed with filter paper and photographed with a camera (Pentax K7) before homogenization.

### Measurement of the Transendothelial Electrical Resistance of VECs

The transendothelial electrical resistance (TER) of VECs in Transwell chambers (Corning Incorporated) was determined with an EVOM2 (Makepolo, USA) instrument (Zhang et al., 2018). Briefly, VECs were inoculated into Transwell chambers with 1×10^5^ cells/well, a small electrode was connected to the upper culture medium, and a larger electrode was connected to the lower culture medium. TER values were dynamically recorded every 15 min. The TER values were normalized to the normal TER.

### Measurement of the Infiltration Rate of FITC-BSA in Monolayer VECs

The FITC-BSA infiltration rate to monolayer VECs was determined as previously described (Zhang et al., 2018), VECs were inoculated into Transwell chambers with 1×10^5^ cells/well, and 200 μL of FITC-BSA (2 mg/ml) was added into the upper compartment. Subsequently, 90 μl of culture medium was collected from the lower compartment at 10, 20, 30, 40, 50, and 60 min after FITC-BSA administration for measurement of the fluorescence intensity with a Synergy HT (BioTek, Winooski, VT, USA) instrument. An equal volume of culture medium was added back to the lower compartment after each collection. The formula for the rate of infiltration of FITC-BSA from the upper compartment to the lower compartment is as follows: infiltration rate of FITC-BSA = {(X1 + X2+…… + Xn)/total fluorescence intensity}×100%, where X1 is the first value, X2 is the second value, and Xn is the nth value. The total fluorescence intensity of 2 mg/ml FITC-BSA was measured at the start of the experiment. The infiltration rate was used to reflect the permeability of VECs.

### Measurement of the Expression of Heparinase, Neuraminidase, Hyaluronidase, VE-Cadherin, Occludin, and Cleaved Caspase-3 by Western Blotting

Western blotting was performed as described previously (Santos-Gallego et al., 2016; Yang et al., 2016), total protein was extracted with RIPA lysis buffer, and protein extracts were separated by SDS-PAGE and then transferred to nitrocellulose membranes and probed with specific antibodies. The band intensities were quantified with an Odyssey CLx (LI-COR) instrument, and the densitometry was determined in Quantity One software (Bio-Rad). Antibodies to the following were used: VE-cadherin (1:1,000, Invitrogen, USA), occludin (1:1,000, Invitrogen, USA), heparinase (1:1,000, Abcam, USA), hyaluronidase (1:1,000, Abcam, USA), neuraminidase (1:1,000, Abcam, USA), cleaved caspase-3 (1:1,000, Cell Signaling Technology, USA), and β-actin (1:7,000, Sigma, USA).

### Immunofluorescence of ZO-1, Heparan Sulfate, Hyaluronic Acid, and Chondroitin Sulfate

Specimens were fixed with 4% paraformaldehyde and permeabilized with 0.2% Triton-X; the specimens were then blocked in 0.1% bovine serum albumin. Antibodies against ZO-1 (1:150, Invitrogen, USA), heparan sulfate (1:150, Abcam, USA), hyaluronic acid (1:150, Abcam, USA), and chondroitin sulfate (1:150, Abcam, USA), or IgG isotype control (1:150, Abcam, USA, **Supplementary Figure 1**) were incubated with the specimens overnight at 4°C. The specimens were then washed with PBS and incubated with Alexa Fluor 488 IgG (Invitrogen, USA) at 37°C for 1 h. Nuclei were stained with DAPI (1:100, Sigma, USA) at 37°C for 20 min. Finally, all samples were imaged with a Leica SP5 confocal microscope (Leica microsystem, Germany). The mean optical density was measured to reflect the content of ZO-1, heparan sulfate, hyaluronic acid, and chondroitin sulfate in endothelial cells.

### Measurement of the Endothelial Glycocalyx by Transmission Electron Microscopy

Transmission electron microscopy methods were as previously described (Fabre-Gray et al., 2018). Rats were anesthetized and perfused with 2% glutaraldehyde, 2% sucrose, 0.1 M sodium cacodylate buffer (pH 7.3), and 2% Alcian blue through a cannula placed in the left ventricle after hemorrhagic shock. Before perfusion, an incision was made in the right atrial appendage, and the neck was ligated with a silk suture. In addition, a perfusion pump was used for injection at a steady rate of 1 ml/min. Thereafter, the lung was harvested and diced. Three or four pieces of approximately 1 mm^3^ each were immersed in perfusion solution for 2 h for fixation and then soaked overnight in a solution without glutaraldehyde before being washed in alkaline (0.03 mol/L NaOH) sucrose (2%) solution. The specimens were then dehydrated through a graded ethanol series. The frozen section method was used to prepare samples; each sample was laid on an iron plate chilled with liquid nitrogen, and ethanol was sprinkled onto the sample. After the ethanol was frozen, the sample was sectioned with a chisel so that it was not touched directly. The samples were then incubated in tert-butyl alcohol at room temperature. After the tert-butyl alcohol had solidified, samples were freeze-dried and examined with TEM (S-4800; Hitachi, Tokyo, Japan).

### Measurement of Leukocyte Adhesion and Neutrophil Infiltration

Leukocyte adhesion was measured as previously described (Han et al., 2009). SD rats were anesthetized and fixed, enterocoelia were opened, mesenteric microvessels (25–40 μm in diameter, 200 μm in length) were exposed and fixed, and the number of adherent leukocytes was observed and recorded under a microcirculation microscope (OLYMPUS, BX51W1, Japan).

The neutrophil infiltration in lung tissue was measured with hematoxylin-eosin (HE) staining. The lung of rats was fixed with 4% formaldehyde through the pulmonary circulation perfusion. The left upper lobe was embedded with paraffin and sliced to 4 μm in thick. HE staining was used to observe the infiltration of neutrophils in alveoli under microscope (Leica DMI 3000B).

### Measurement of Renal Function and Coagulation, and Levels of TNF-α and IL-6 in Rats

Blood samples were collected from SD rats. Renal function was measured with biochemical analyzer (LX-20, Beckman Coulter Inc., Brea, CA, USA). Coagulation function was measured with ACL 200 blood coagulation analyzer according to the instructions of the HemosIL kit.

The blood TNF-α and IL-6 were quantified with TNF-α (Diaclone Research, Besancon, France) and IL-6 (Biosource Europe SA, Niveiles, Belgium) ELISA kits according to the manufacturers' instructions.

### Statistical Analysis

All data are presented as mean ± SD. The differences in data among groups were analyzed by one-way ANOVA when normality (homogeneity of variance) assumptions were satisfied; otherwise, the equivalent non-parametric test was used with SPSS version 19.0 package (IBM). P <0.05 was considered significant.

## Results

### Effect of HES (130/0.4) on Pulmonary Vascular Permeability After Hemorrhagic Shock

**Figure 2:**
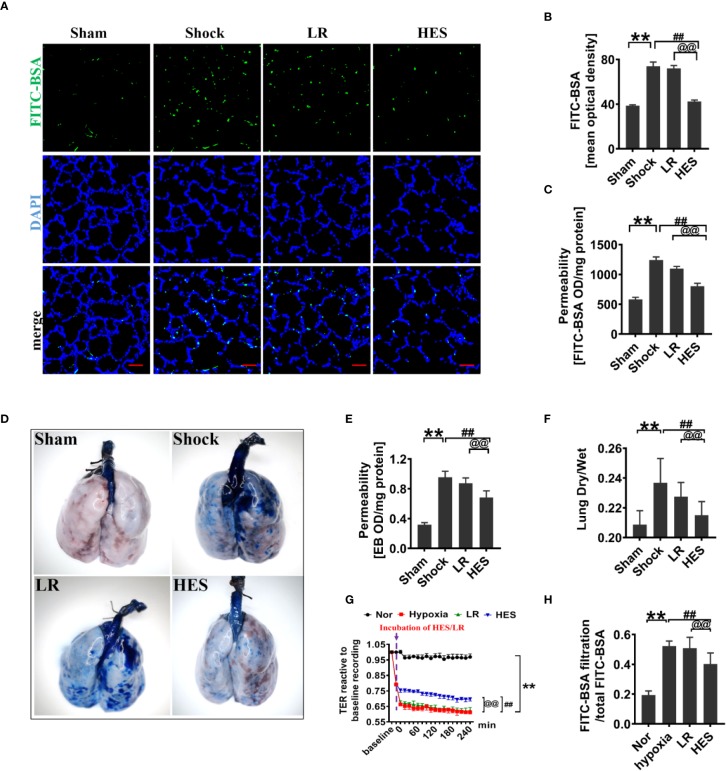
Protective effect of HES (130/0.4) on vascular permeability after hemorrhagic shock (n = 8). **(A, B)**. Vascular permeability of the lung, measured by the mean optical density of intravenously injected of FITC-BSA *in vivo* (bar = 50 μm). **(C)** Vascular permeability of the lung, measured by the fluorescence optical density (OD) value of FITC-BSA in lung homogenate. **(D, E)** Vascular permeability of the lung, measured by the leakage of Evans blue. **(F)** Measurement of the dry/weight ratio of the lung after hemorrhagic shock and HES (130/0.4) treatment. **(G, H)** Effect of HES (130/0.4) on the transendothelial electrical resistance (TER) and infiltration rate of FITC-BSA in monolayer VECs after hypoxic treatment. Data are presented as mean ± SD; **P < 0.01, as compared with the sham operated/normal group; ^##^P < 0.01, as compared with the shock/hypoxia group, ^@@^P < 0.01, as compared with the LR group. Sham, sham operated group; Nor, normal group; HES, hydroxyethyl starch; LR, lactated Ringer's solution; FITC-BSA, fluorescein isothiocyanate-labeled bovine albumin V; TER, transendothelial electrical resistance; VECs, vascular endothelial cells.

To assess the effect of HES (130/0.4) on pulmonary vascular permeability after hemorrhagic shock, a rat model was used (**Figure 1**). The results showed that the pulmonary vascular permeability was significantly increased after hemorrhagic shock. It appeared the vascular leakage of FITC-BSA was significantly higher in the hemorrhagic shock group than that in sham operated group (**Figures 2A, B**). The leakage of Evans blue in lung tissue in hemorrhagic shock group was also greater than that in sham operated group (**Figures 2D, E**). Meanwhile, the fluorescence OD value of FITC-BSA in lung tissue in hemorhagic shock group was significantly higher than that in sham operated group (**Figure 2C**). LR infusion did not decrease the leakage of FITC-BSA and Evans blue induced by hemorrhagic shock (**Figures 2A–E**). HES (130/0.4) infusion significantly decreased hemorrhagic shock induced pulmonary vascular leakage, it appeared the fluorescence exudation of FITC-BSA in lung tissue was significantly lower than that in LR group. The fluorescence density of FITC-BSA in lung tissue was also significantly lower than that in LR group. Meanwhile, the OD value of Evans blue in hemorrhagic shock group was significantly lower than that in LR group (**Figures 2A–E**). Also, the dry/wet weight ratio of lung in hemorrhagic shock group was significantly higher than that in sham operated group. HES (130/0.4) infusion could decrease the dry/wet weight ratio of lung after hemorrhagic shock while LR infusion did not (**Figure 2F**).

To further explore the effect of HES (130/0.4) on the permeability of VECs, hypoxia treated VECs was used to incubate with 1% of HES (130/0.4) or LR for 2 h, and the changes of TER and FITC-BSA infiltration rate were observed. The results found that the TER of VECs was significantly decreased, and the FITC-BSA infiltration rate of monolayer VECs was significantly increased after hypoxia as compared to the normal group (**Figures 2G, H**). LR incubation only slightly improved the TER of VEC and FITC-BSA infiltration rate after hypoxia, while HES (130/0.4) incubation resulted in a significant increase in TER and a significant decrease in FITC-BSA infiltration rate (**Figures 2G, H**).

### Role of Endothelial Glycocalyx in HES Protecting Vascular Permeability After Hemorrhagic Shock

**Figure 3:**
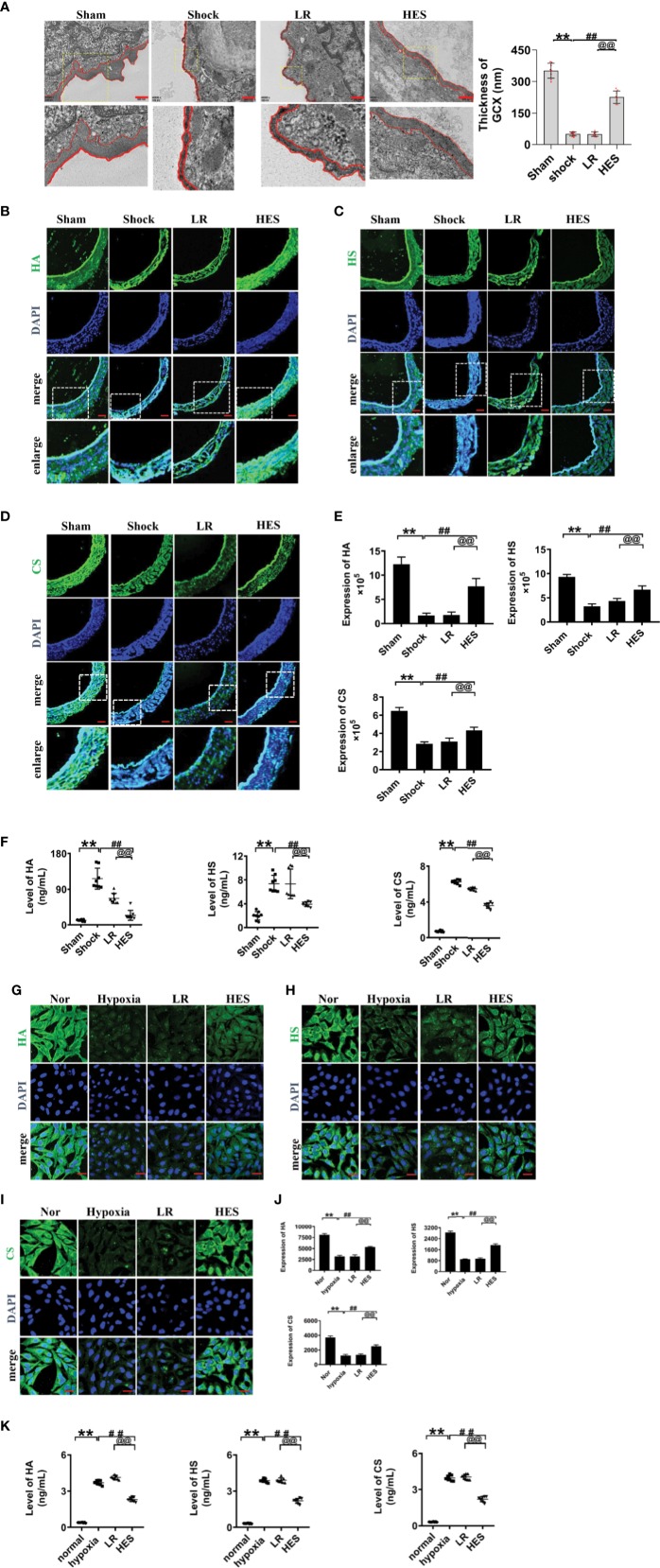
Effects of HES (130/0.4) on the expression of endothelial glycocalyx components in the pulmonary vein after hemorrhagic shock and in VECs after hypoxia (n = 8). **(A)** Measurement of the structure of endothelial glycocalyx in pulmonary vein after hemorrhagic shock, determined by transmission electron microscopy. The endothelial glycocalyx was enhanced with Alcian blue staining before fixation (bar=500 nm). **(B–E)**. Measurement of the expression of hyaluronic acid (HA, green), heparan sulfate (HS, green), and chondroitin sulfate (CS, green) in pulmonary vein after hemorrhagic shock (bar=50 μm). **(F)** Measurement of levels of hyaluronic acid, heparan sulfate, and chondroitin sulfate in the plasma with an ELISA kit. **(G–J)**. Measurement of the expression of hyaluronic acid (HA, green), heparan sulfate (HS, green), and chondroitin sulfate (CS, green) in VECs after hypoxic treatment (bar=25 μm). Data are presented as mean ± SD; **P < 0.01, as compared with the sham operated group/normal group; ^##^P < 0.01, as compared with the shock group/hypoxia group; ^@@^P < 0.01, as compared with the LR group. Sham, sham operated group; Nor, normal group; HES, hydroxyethyl starch; LR, lactated Ringer's solution; GCX, glycocalyx; HA, hyaluronic acid; HS, heparan sulfate; CS, chondroitin sulfate; VECs, vascular endothelial cells.

Previous studies have found that endothelial glycocalyx plays an important role in vascular barrier integrity. To clarify the role of endothelial glycocalyx in the beneficial effect of HES (130/0.4) on pulmonary vascular permeability after hemorrhagic shock, we examined the changes of endothelial glycocalyx in rats after hemorrhagic shock and the effect of HES (130/0.4) or LR infusion. The results showed that the structure of endothelial glycocalyx in pulmonary vein was damaged after hemorrhagic shock. The thickness of endothelial glycocalyx (351.6 nm) in sham operated group was significantly greater than in shock group (50.7 nm). LR infusion did not improve the endothelial glycocalyx in thickness (50.0 nm) (**Figure 3A**), while HES (130/0.4) infusion effectively ameliorated the structure of endothelial glycocalyx. The thickness of endothelial glycocalyx reached to 226.2 nm, 3.5 times greater than that in the LR group (**Figure 3A**).

The expression of heparan sulfate, hyaluronic acid, and chondroitin sulfate in endothelial glycocalyx after hemorrhagic shock was further detected, and the results showed that the expression of the components of endothelial glycocalyx was significantly decreased after hemorrhagic shock. LR infusion did not significantly improve the expression of hyaluronic acid, heparan sulfate and chondroitin sulfate. While HES infusion significantly increased the expression of the components of endothelial glycocalyx. (**Figures 3B–E**).

The components of the destroyed endothelial glycocalyx can enter into the blood, and therefore, the concentrations of endothelial glycocalyx components in plasma partly reflect the severity of injury (Johansson et al., 2012; Ostrowski et al., 2015). Our results found the concentration of hyaluronic acid, heparan sulfate, and chondroitin sulfate in plasma was significantly increased after hemorrhagic shock as compaered to sham operated group. LR infusion did not affect the plasma concentration of hyaluronic acid, heparan sulfate, and chondroitin sulfate as compared to hemorrhagic shock group. While HES (130/0.4) infusion significantly decreased the plasma concnetration of hyaluronic acid, heparan sulfate, and chondroitin sulfate (**Figure 3F**).

Furthermore, our results found that the expression of hyaluronic acid, heparan sulfate, and chondroitin sulfate in cultured VEC was also signifcanly decreased after hypoxia. LR incubation did not change the expression of endothelial glycocalyx components of hypoxia treated VECs, while HES (130/0.4) incubation resulted in the significant recovery in the expression of endothelial glycocalyx components (**Figures 3G–J**). Culture medium measurement found that the concentration of HA, HS, and CS in culture medium of VECs was significantly increased after hypoxia. LR incubation did not affect the increase of HA, HS, and CS in culture medium, while HES incubation significantly decreased the concentration of HA, HS, and CS in culture medium of VECs (**Figure 3K**). These results indicated that the endothelial glycocalyx was destroyed after hemorrhagic shock/hypoxia, and HES (130/0.4) had beneficial effect to the endothelial glycocalyx.

**Figure 4:**
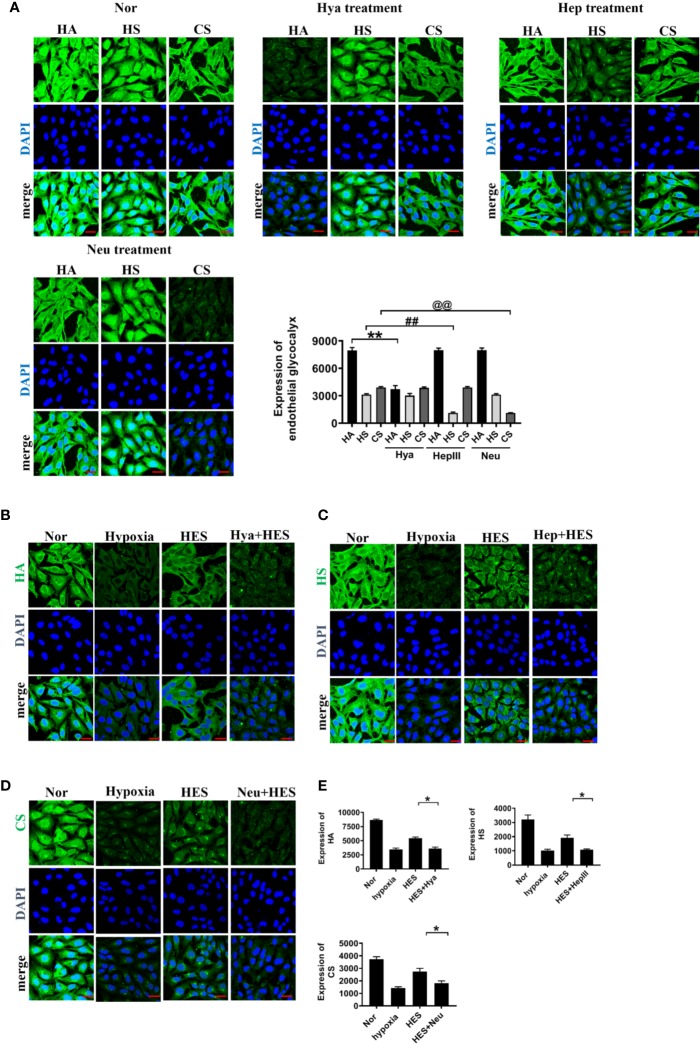
The role of endothelial glycocalyx degrading enzymes in the protective effect of HES (130/0.4) on the endothelial glycocalyx (n = 8). **(A)** Effects of endothelial glycocalyx degrading enzymes on the expression of endothelial glycocalyx (bar=25 μm). Data are presented as mean ± SD; **P < 0.01, as compared with the HA group; ^##^P < 0.01, as compared with the HS group; ^@@^P < 0.01, as compared with the CS group. **(B–E)**. The role of endothelial glycocalyx degrading enzymes on the recovery effect of endothelial glycocalyx by HES (130/0.4) (bar = 25 μm). Expression of HA (**B**, green), HS (**C**, green), CS (**D**, green) in VECs after treatment with endothelial glycocalyx degrading enzymes and hypoxia. Data are presented as mean ± SD; *P < 0.05, as compared with the HES (130/0.4) group. HES, hydroxyethyl starch; Nor, normal group; Hya, hyaluronidase; Hep, heparinase III; Neu, neuraminidase; HA, hyaluronic acid; HS:heparan sulfate; CS,chondroitin sulfate.

**Figure 5:**
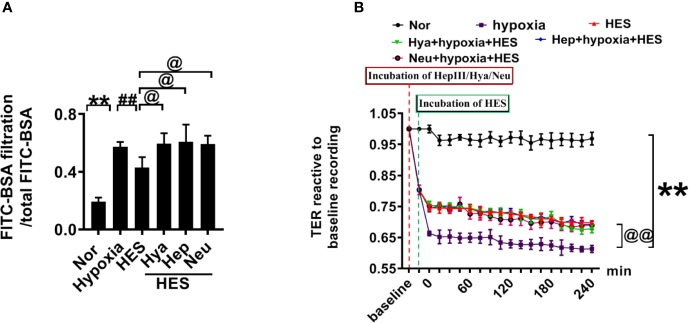
The role of endothelial glycocalyx degrading enzyme in the protective effect of HES (130/0.4) on VEC permeability. **(A, B)**. Effects of endothelial glycocalyx degrading enzymes on the transendothelial electrical resistance (TER) and the infiltration rate of FITC-BSA in monolayer VECs after hypoxia and HES (130/0.4) treatment. Data are presented as mean ± SD; **P < 0.01, as compared with the normal group; ^@^P < 0.05, ^@@^P < 0.01, as compared with the hypoxia group; ^##^P < 0.01, as compared with the HES (130/0.4) group. Nor, normal group; HES, hydroxyethyl starch; Hya, hyaluronidase; Hep, heparinase III; Neu, neuraminidase; VECs, vascular endothelial cells.

To clarify how HES protect the endothelial glycocalyx after hemorrhagic shock, we observed the effects of endothelial-glycocalyx-degrading enzymes on the protective effect of HES on vascular permeability in cultured VEC. VECs were resectively incubated with endothelial glycocalyx degrading enzyme (heparinase III: 2 U/ml, hyaluronidase: 25 U/ml, and neuraminidase: 0.83 U/ml in serum-free medium at 37°C for 6 h) (Mockl et al., 2017), the degrading effect of endothelial glycocalyx was observed. The results showed that the endothelial-glycocalyx-degrading enzymes significantly inhibited the expression of hyaluronic acid, heparan sulfate, and chondroitin sulfate in VEC. Each degrading enzyme had no cross-reactivity with other endothelial glycocalyx components. The results suggest that each endothelial glycocalyx degrading enzyme effectively degraded the corresponding components (**Figure 4A**). Further study found that endothelial-glycocalyx-degrading enzymes inhibited the recovery effect of HES (130/0.4) on the expression of hyaluronic acid, heparan sulfate, and chondroitin sulfate (**Figures 4B–E**), and antagonized the protective effect of HES on VEC permeability, it appeared the infiltration rate of FITC-BSA to VEC was significantly increased and the TER was decreased (**Figures 5A, B**). The results indicated that HES (130/0.4) improving VEC permeability is mainly through endothelial glycocalyx degrading enzyme.

**Figure 6:**
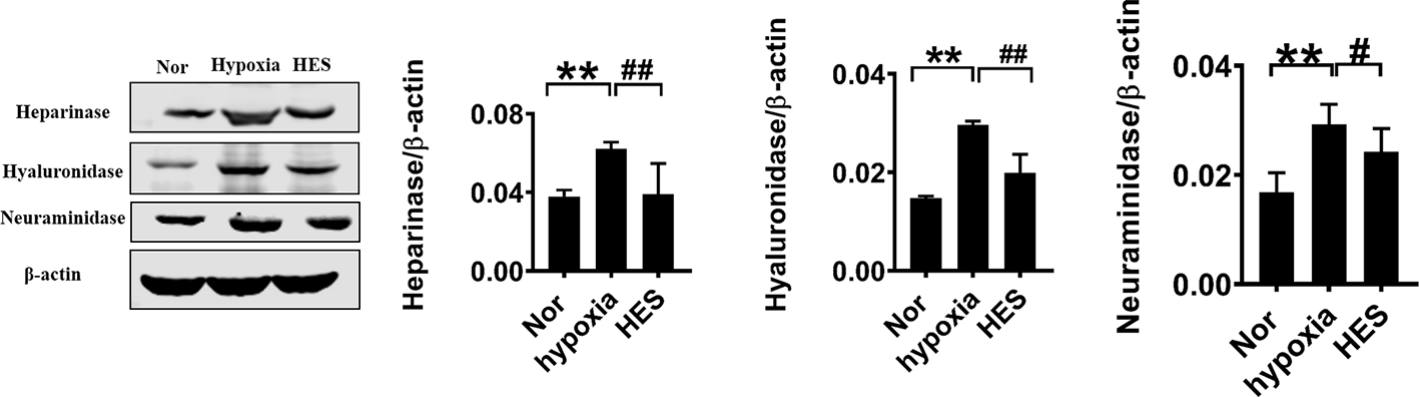
Effect of HES (130/0.4) on the expression of heparinase, hyaluronidase, neuraminidase in VECs after hypoxia (n = 8). Data are presented as mean ± SD; **P < 0.01, as compared with the normal group; ^#^P < 0.05, ^##^P < 0.01, as compared with the hypoxia group. Nor, normal group; HES, hydroxyethyl starch; VECs, vascular endothelial cells.

To further determine the relationship of HES (130/0.4) protecting the vascular permeability and endothelial glycocalyx degrading enzymes, we examined the effect of HES (130/0.4) on the expression of the endothelial glycocalyx degrading enzymes heparinase, hyaluronidase, and neuraminidase in VECs after hypoxia. The results showed that the expression of heparinase, hyaluronidase, and neuraminidase in VECs was significantly increased after hypoxia. In contrast, HES (130/0.4) incubation significantly decreased the expression of heparinase, hyaluronidase, and neuraminidase (**Figure 6**). The results indicated that HES (130/0.4) protected the endothelial glycocalyx mainly through down-regulation of the expression of heparinase, hyaluronidase, and neuraminidase.

### Effect of HES (130/0.4) on Intercellular Junction Proteins in Pulmonary Vein After Hemorrhagic Shock and Its Relationship With Endothelial Glycocalyx

**Figure 7:**
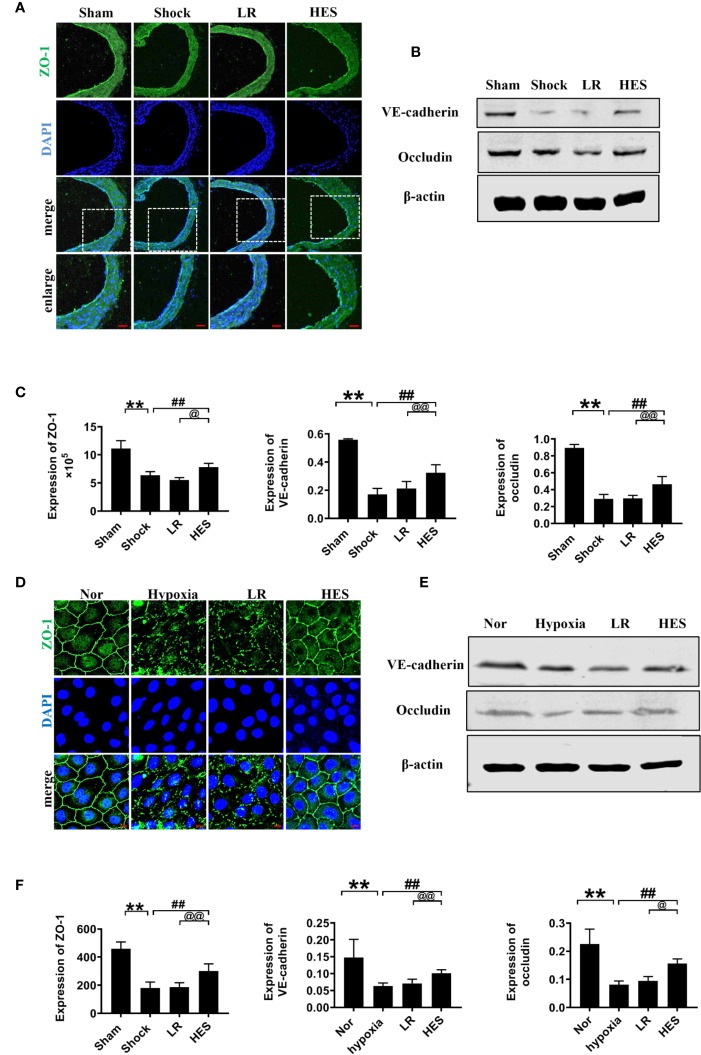
Effects of HES (130/0.4) on the expression of intercellular junction proteins after hemorrhagic shock/hypoxia (n = 8). **(A)** Measurement of the expression of ZO-1 (green) after hemorrhagic shock in pulmonary vein by immunofluorescence (bar = 50 μm). **(B)** Measurement of the expression of VE-cadherin and occludin after hemorrhagic shock in the pulmonary vein, determined by western blotting. **(C)** Statistical diagram of the expression of ZO-1, VE-cadherin, and occludin in the pulmonary vein *in vivo*. **(D)** Measurement of the expression of ZO-1 (green) after hypoxia treatment (bar = 10 μm). **(E)** Measurement of the expression of VE-cadherin and occludin after hypoxia treatment. **(F)** Statistical diagram of the expression of ZO-1, VE-cadherin, and occludin in VECs *in vitro*. Data are presented as mean ± SD; **P < 0.01, as compared with the sham operated group/normal group; ^##^P < 0.01, as compared with the shock/hypoxia group; ^@^P < 0.05, ^@@^P < 0.01, as compared with the LR group. Nor, normal group; Sham, sham operated group; HES, hydroxyethyl starch; LR, lactated Ringer's solution; VECs, vascular endothelial cells.

Intercellular junction proteins play important role in vascular permeability. To measure the effect of HES (130/0.4) on intercellular junction proteins in VECs after hemorrhagic shock, we measured the expression of intercellular junction proteins ZO-1, VE-cadherin, and occludin in pulmonary vein in hemorrhagic shock rat and hypoxia treated VEC. The expression of ZO-1, VE-cadherin, and occludin in the pulmonary vein was significantly decreased after hemorrhagic shock, LR infusion slightly increased the expression of intercellular junction proteins. While HES (130/0.4) significantly increased the expression of ZO-1, VE-cadherin, and occludin (**Figures 7A-C**). In addition, the expression of ZO-1, VE-cadherin, and occludin in hypoxic VECs was significantly lower than that in the normal group (**Figures 7D–F**). The expression of intercellular junction proteins was increased slightly after incubation with LR. HES (130/0.4) incubation significantly increased the expression of ZO-1, VE-cadherin, and occludin in hypoxia-treated VEC (**Figures 7D–F**).

**Figure 8:**
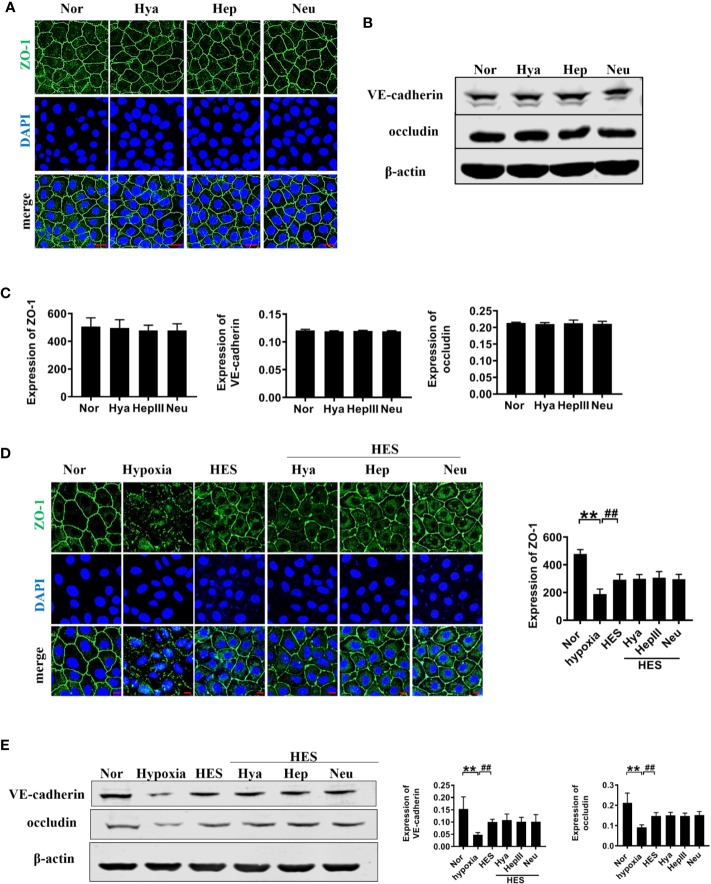
The role of endothelial glycocalyx degrading enzymes in the protective effects of HES (130/0.4) on intercellular junction proteins. **(A–C)**. Effects of endothelial glycocalyx degrading enzymes on the expression of ZO-1, VE-cadherin, and occludin in VECs *in vitro*, and statistical diagram (bar = 25 μm). **(D, E)**. The roles of endothelial glycocalyx degrading enzymes in the recovery effect of HES on the expression of ZO-1, VE-cadherin, and occludin, and statistical diagram (bar = 10 μm). Data are presented as mean ± SD; **P < 0.01, as compared with the normal group; ^##^P < 0.01, as compared with the hypoxia group. Nor, normal group; HES, hydroxyethyl starch; Hya, hyaluronidase; Hep, heparinase III; Neu, neuraminidase; VECs, vascular endothelial cells.

To further clarify the relationship between the recovery effect of HES (130/0.4) on intercellular junction proteins and endothelial glycocalyx after hemorrhagic shock, we examined the expression of intercellular junction proteins in VECs after incubation with endothelial-glycocalyx-degrading enzymes. The expression of ZO-1, VE-cadherin, and occludin in pulmonary vein did not change significantly after use of endothelial-glycocalyx-degrading enzymes in hemorrhagic shock rats (**Figures 8A–C**). Simultaneously, use of endothelial glycocalyx degradation enzymes did not affect HES (130/0.4) induced increase of the expression of the intercellular junction proteins ZO-1, VE-cadherin, and occludin (**Figures 8D–F**). Results suggests that the protective effect of HES (130/0.4) on intercellular junction proteins in hemorrhagic shock was not associated with the endothelial glycocalyx.

### Effects of HES (130/0.4) on Inflammation, Coagulation Function, Renal Function, and Apoptosis After Hemorrhagic Shock

**Figure 9:**
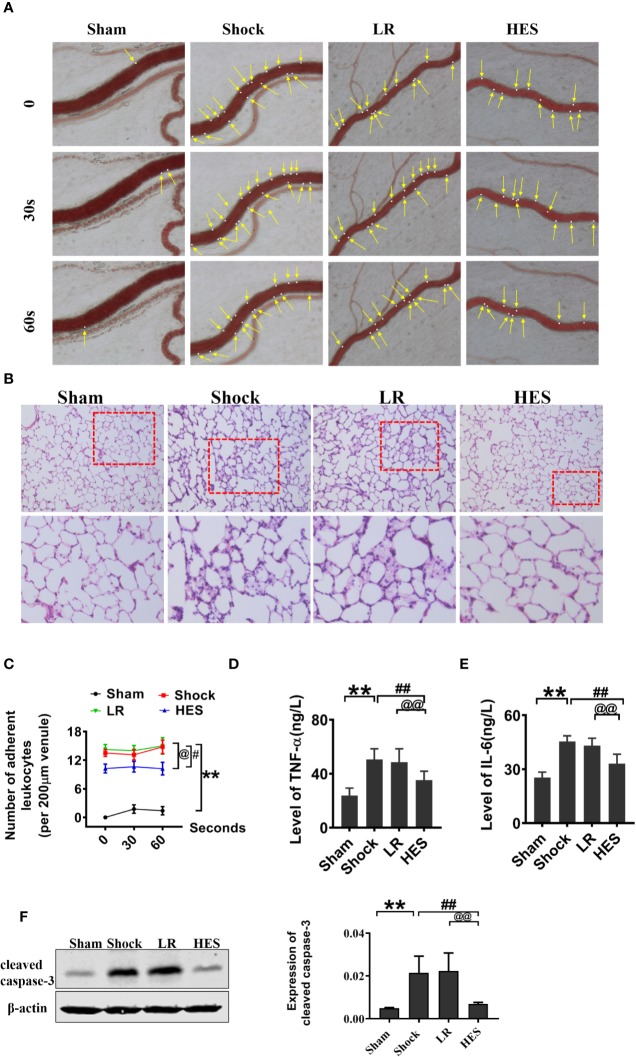
Effects of HES (130/0.4) on leucocyte adhesion, neutrophil recruitment, and inflammation after hemorrhagic shock (n = 8). **(A, C)**. Effect of HES (130/0.4) on the adhesion of leukocytes in mesenteric venules after hemorrhagic shock (bar = 50 μm). **(B)** Effects of HES (130/0.4) on the recruitment and infiltration of neutrophils in lung tissue (bar = 50 μm). **(D, E)**. Effects of HES (130/0.4) on inflammation after hemorrhagic shock. **(F)** Effects of HES (130/0.4) on apoptosis of VECs in the pulmonary vein after hemorrhagic shock. Data are presented as mean ± SD; **P < 0.01, as compared with the sham operation group; ^#^P < 0.05, ^##^P < 0.01, as compared with the shock group; ^@^P < 0.05, ^@@^P < 0.01, as compared with the LR group. HE-staining, hematoxylin-eosin staining; ELISA, enzyme linked immunosorbent assay; Sham, sham operated group; HES, hydroxyethyl starch; LR, lactated Ringer's solution.

Because inflammation, coagulation dysfunction, and renal dysfunction are important complications of hemorrhagic shock and HES (130/0.4) infusion, we examined the changes in inflammation, coagulation, and renal function after HES (130/0.4) treatment. The number of adherent leukocytes in mesenteric venules, and the concentration of plasma TNF-α and IL-6 significantly increased after hemorrhagic shock. LR infusion could slightly decrease TNF-α and IL-6 concentration in plasam. HES (130/0.4) infusion significantly decreased the number of adherent leukocytes and TNF-α and IL-6 concentration (**Figures 9A, C–E**). Pathological examination revealed neutrophil infiltration increase and alveolar wall thickening in lung tissue after hemorrhagic shock, and there was no improvement in pathological changes in lung tissue after LR infusion. HES (130/0.4) infusion significantly ameliorated the infiltration of neutrophils in lung tissue after hemorrhagic shock (**Figure 9B**).

The effect of HES on apoptosis after hemorrhagic shock was also observed, and the results indicated that the expression of cleaved caspase-3 in the pulmonary vein was significantly increased after hemorrhagic shock. LR did not improve the expression of cleaved caspase-3. As compared with LR, HES (130/0.4) significantly decreased the expression of cleaved caspase-3 (**Figure 9F**).

**Figure 10:**
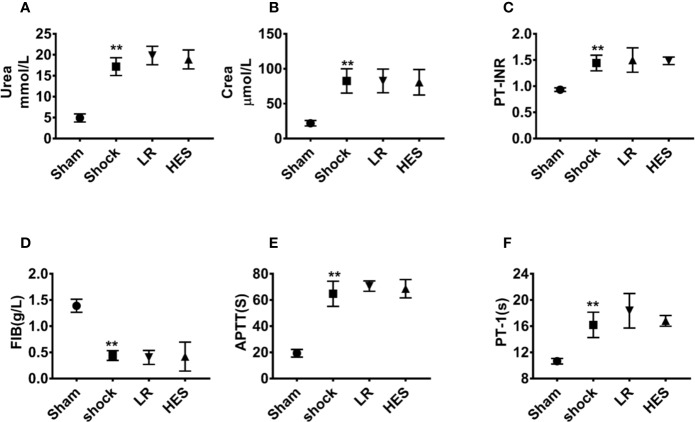
Effects of HES (130/0.4) on renal and coagulation functions in the blood of rats with hemorrhagic shock (n = 8). **(A, B)** Effect of HES (130/0.4) on renal function in the blood of rats with hemorrhagic shock. **(C–F)** Effect of HES (130/0.4) on coagulation function in the blood of rats with hemorrhagic shock. Data are presented as mean ± SD; **P < 0.01, as compared with the sham operated group. PT-INR, prothrombin time international normalized ratio; FIB, fibrinogen; APTT, activated partial thromboplastin time; PT-1, prothrombin time; Sham, sham operated group; HES, hydroxyethyl starch; LR, lactated Ringer's solution.

Further studies showed that the levels of urea and Crea in plasma were significantly increased after hemorrhagic shock as compared with those in the sham operation group. The levels of PT-INR, APTT, and PT-1 were also significantly increased, whereas the level of FIB was significantly decreased. There were no significant changes in the levels of urea, Crea, PT-INR, APTT, PT-1, and FIB in the blood after infusion of LR or HES (130/0.4) (**Figures 10A–F**). These results indicated that HES (130/0.4) ameliorated inflammation after hemorrhagic shock, and the dose of HES (130/0.4) used in the present study did not aggravate renal and coagulation dysfunction after hemorrhagic shock.

## Discussion

Hemorrhagic shock and fluid resuscitation are often accompanied by severe vascular leakage, which will lead to organ dysfunction. Exploring effective measures for preventing and treating vascular leakage is particularly important for the treatment of hemorrhagic shock. The present study indicated that pulmonary vascular permeability was increased after hemorrhagic shock. HES (130/0.4) protected hemorrhagic shock induced pulmonary vascular leakage by protecting the endothelial glycocalyx and intercellular junction proteins. HES (130/0.4) protecting the structure and the expression of endothelial glycocalyx is mainly through down-regulation of the expression of endothelial glycocalyx degradation enzyme heparinase, hyaluronidase, and neuraminidase. HES (130/0.4) also had anti-inflammatory and anti-apoptotic effects in hemorrhagic shock.

Fluid resuscitation is an important measure to improve tissue perfusion and maintain hemodynamic stability after hemorrhagic shock. HES has been shown to have an efficacious blood volume expansion effect (Myburgh et al., 2012; van Galen and Hallowell, 2019), and hemodynamics stabilization effect. Kheirabadi et al. found that 6% HES (130/0.4) improved the hemodynamic parameters of New Zealand white rabbits in uncontrolled hemorrhagic shock (Kheirabadi et al., 2017). HES was also found to be able to improve the oxygen transport and the serum lactic acid clearance rate in a dog model of hemorrhagic shock (McBride et al., 2017; Wu et al., 2019). In addition, HES was found to have a good effect on improving organ function, and anti-inflammatory effects. Balkamou found that HES (130/0.4) could regulate the production and release of inflammatory cytokines, and improve acute lung injury in swine hemorrhagic shock (Balkamou et al., 2010). Varga found that as compared with 4% gelatin and 6% dextran solution, 6% HES 130/0.4 provided a therapeutic advantage in ischemia-reperfusion by exerting an inhibitory effect on ischemia reperfusion-induced local and systemic leukocyte reactions and post-ischemic periosteal microvascular dysfunction in rats (Varga et al., 2008). Rossaint confirmed that HES 130/0.4 can decrease neutrophil recruitment in the lung, liver, and kidney in mice with systemic inflammation (Rossaint et al., 2015). Moreover, some studies found that HES has anti-apoptotic functions in hemorrhagic shock. Tsai found that HES (130/0.4) inhibited the increase in the Bcl-2/Bax ratio induced by hemorrhagic shock and improved the anti-apoptosis ability of the heart and lung in rats (Tsai et al., 2007). Liang also found that HES (130/0.4) prevented bone marrow mononuclear cell apoptosis in a rat model with hemorrhagic shock (Liang et al., 2010). In the present study, we found that HES exerted an anti-apoptotic effect by down-regulating the expression of cleaved-caspase-3.

However, the therapeutic effects of HES with different molecular weight are different. Standl et al. found that HES 130/0.4 resulted in a more pronounced and earlier increase in skeletal muscle tpO(2), in comparison with pre-hemodilution values, than HES 70/0.5 or HES 200/0.5 (Standl et al., 2003). Cui et al. reported that, in patients undergoing liver surgery involving extensive blood loss, HES (130/0.4), as compared with HES (200/0.5), resulted in a greater improvement in internal organ perfusion and tissue oxygenation (Cui et al., 2014). Simultaneously, the effect of HES on vascular permeability is also controversial. Some studies suggested that HES has a positive protective effect on vascular leakage. Zikria et al. reported that HES macromolecules (100–300 kDa) repaired barrier damage by physically covering the sites of barrier damage in a rat model of standardized scald burns (Zikria et al., 1989). Tian et al. found that 6% HES 200/0.5 attenuated microvascular leakage by inhibiting the recruitment and infiltration of inflammatory mediators and neutrophils in endotoxin-induced sepsis rats (Tian et al., 2004). Other studies found the opposite effects of HES on vascular leakage. Wong et al. found that HES (130/0.4, 3%) increased the permeability of human umbilical vein endothelial cells and epithelial cells, thus, resulting in significant increase of transmembrane transfer of FITC-dextran *in vitro* (Wong et al., 2019). They also found that HES (130/0.4) infusion increased the transfer of FITC-dextran from blood vessels to the lumen in an isolated small intestine perfusion model of mice *in vivo* (Wong et al., 2016). Some researchers believed that, because HES is osmotically active and is able to bind large amount of water, the fluid shift from blood vessel to interstitial compartment might be increased, thus leading to a transient interstitial edema (Wiedermann and Joannidis, 2014). In the present study, we found that HES attenuated the vascular leakage induced by hemorrhagic shock by protecting the endothelial glycocalyx.

The endothelial glycocalyx is a dynamic structure covering the endothelial surface. Many studies found that some pathophysiological processes are associated with structural and functional disorder of endothelial glycocalyx (Salmon and Satchell, 2012; Alphonsus and Rodseth, 2014; Pillinger and Kam, 2017), which serves as an endothelial barrier. Previous studies confirmed that endothelial glycocalyx played an important role in endothelial cell permeability (Alphonsus and Satchell, 2014b, Curry, 2018; Ma et al., 2019). Torres et al. found that hemorrhagic shock (40% blood loss) decreased the thickness of endothelial glycocalyx in skeletal muscle venules by 59% (Torres et al., 2013; Torres et al., 2017). Margraf et al. found that the decreased expression of endothelial glycocalyx components in pulmonary vessels in sepsis mice resulted in the increase of vascular permeability (Margraf et al., 2018). Salmon et al. found that the damage of endothelial glycocalyx in structure also increased the vascular permeability in experimental trauma and ischemia-reperfusion injury (Henrich et al., 2010; Salmon and Satchell, 2012; Myburgh and Mythen, 2013). Halbagebauer et al. found that in hemorrhagic shock with polytrauma, the concentration of heparan sulfate in plasma was significantly increased (Halbgebauer et al., 2018). In the present study, we found that the structure of endothelial glycocalyx in pulmonary vein was destroyed; the expression of heparan sulfate, hyaluronic acid, and chondroitin sulfate was decreased after hemorrhagic shock in a rat model; and HES (130/0.4) resulted in the recovery of endothelial glycocalyx of rats with hemorrhagic shock. Previous studies also reported that HES has a protective effect on the endothelial glycocalyx. Margraf et also found that 6% HES (130/0.4) has a protective effect on the integrity of pulmonary vein endothelial glycocalyx in systematic and pulmonary information mouse model (Margraf et al., 2018). Moreover, Job et al. found that 0.1–1% HES (600/0.75) has a dose-dependent protective effect on the endothelial glycocalyx (Job et al., 2016).

In the present study, we also found that HES (130/0.4) protecting endothelial glycocalyx was mainly by down-regulating the expression of heparinase, hyaluronidase, and neuraminidase. However, how HES (130/0.4) acting on theses degrading enzymes has not been studied. Previous studies found that overexpression of inflammatory cytokines and ROS/RNS can lead to abnormal expression of endothelial glycocalyx-related enzymes. Kolarova et al. found that the overexpression of TNF-α activates the expression of heparinase and induced the degradation of the endothelial glycocalyx in pulmonary microvessels in endotoxin treated mice (Kolarova et al., 2014). The increase in ROS in both glomerular endothelial cells and epithelial cells can increase the expression of heparinase, which degrades heparan sulfate (Kramer et al., 2006; Rao et al., 2011). Tsai et al. found that HES (10%) infusion eliminated the ROS burst induced by hemorrhagic shock (Tsai et al., 2007). Whether HES protecting the endothelial glycocalyx by decreasing ROS and the expression of degrading enzymes needs to be further studied. Previous studies showed that ROS/RNS induced modification of glycosyl residues of endothelial glycocalyx, and destroyed the original molecular structure of endothelial glycocalyx; therefore, resulting in the formation of unstable intermediates, which is easy to be degraded into small molecule through hydrolysis (van Golen et al., 2012). HES molecule contains more active hydroxyl groups, which can easily form stable hydrogen bonds with other molecules. Therefore, we speculate that hydroxyl groups in HES may form stable covalent bonds or hydrogen bonds with endothelial glycocalyx molecules, thus, increases the resistance ability of endothelial glycocalyx molecules to ROS and other molecules. In addition, HES can be hydrolyzed by α-amylase *in vivo* and eventually metabolized into monosaccharide, which participates in glycometabolism reactions. Monosaccharides in the Golgi apparatus in endothelial cells can be used as substrates for the synthesis of endothelial glycocalyx and participate in the synthesis of the endothelial glycocalyx (Becker et al., 2010). HES may participate in the synthesis of the endothelial glycocalyx by metabolizing monosaccharides, although the specific mechanism requires further study.

In the present study, we found that HES (130/0.4) protected vascular permeability not only by protection of the endothelial glycocalyx, but also by protecting the intercellular junction proteins such as ZO-1. Furthermore, the experimental results found that the protective effect of HES on the expression of intercellular junction protein ZO-1, VE-cadherin, and occludin *in vitro* was better than *in vivo* levels. The reasons for this difference may be: *In vitro*, the concentration of HES with effect in cells may be higher than that *in vivo*. At the same time, in vascular endothelial cells *in vitro*, the concentration and types of molecules that have degradation effect on HES are less than that *in vivo*, and the degradation and metabolism effect of HES is relatively low. However, the effect of HES (130/0.4) on the expression of intercellular junction was not related to the endothelial glycocalyx, and how HES protecting intercellular junction proteins and the detailed relationship among HES, endothelial glycocalyx and intercellular junction proteins need further investigation.

Previous studies showed that myosin light chain kinase (MLCK) and myosin light chain phosphatase (MLCP) play important role in the regulation of vascular leakage. Under hypoxia, the Ca^2+^/CaM-PKC (protein kinase C) pathway and Ca^2+^-cGMP-PKG (protein kinase G) pathway, which are mediated by up-regulation of intracellular Ca^2+^, could in turn upregulate the phosphorylation of MLC by activating MLCK, thus, resulting in abnormal connexins and increased vascular permeability (Duan et al., 2017). In addition, Rose et al. found that HES could decrease the influx of Ca^2+^ in hepatocytes after hemorrhagic shock in rats (Rose et al., 2000). Thus, we speculate that HES (130/0.4) playing anti-leakage role in vascular permeability after hemorrhagic shock might also be *via* inhibiting Ca^2+^ influx into cells, and then blocking the activation of the Ca^2+^/CaM-PKC pathway and Ca^2+^-cGMP-PKG pathway, except for protecting endothelial glycocalyx and intercellular junction proteins.

With the wide application of HES in fluid resuscitation therapy, an increasing number of studies showed that HES had adverse effects on coagulation and renal function (Svensen and Hahn, 1997; de Jonge and Levi, 2001; Schortgen et al., 2001; Johansson et al., 2011). Haase et al. found that HES can easily increase the burden of the kidney, leading to renal dysfunction, and increase the probability of erythrocytic transfusion (Haase et al., 2013), and it is also not conducive to improving 90-day survival rates in patients with sepsis (Serpa et al., 2014; Leal et al., 2017). In a human study for the side effects of HES, transfusion with HES increased the risk of bleeding and the need for blood products (Finfer et al., 2010). Our present study found that 2% HES had no side effects on coagulation and renal function, it neither improved nor aggravated the coagulation or renal dysfunction induced by hemorrhagic shock. These findings may be associated with the low molecular weight (molecular weight 130 kD) and low concentration of HES used herein. HES can be hydrolyzed into small molecules and metabolized out of the body without aggravating the burden of kidney and coagulation function (Momeni et al., 2017).

## Conclusions

HES (130/0.4) improves pulmonary vascular permeability after hemorrhagic shock mainly through protecting endothelial glycocalyx and intercellular junction proteins. The protective effect of HES (130/0.4) on endothelial glycocalyx is mainly through HES down-regulation of the expression of endothelial glycocalyx degradation enzymes. The protective effect of HES (130/0.4) on intercellular junction proteins was not associated with endothelial glycocalyx.

### Limitations

In the present study, the results found that HES could protect the vascular permeability of hemorrhagic shock through endothelial glycocalyx and intercellular junction. The mechanism still needs to be studied by experiments. As for the mechanism of HES protecting intercellular junction, we will explore the effect of HES on the upstream molecules of intercellular junction pathway at the protein level and transcription level, such as myosin light chain kinase, myosin light chain phosphatase, and further clarify the mechanism of HES protecting intercellular junction; in the aspect of HES protecting endothelial glycocalyx, the present study found that HES can protect endothelial glycocalyx by down regulating endothelial glycocalyx degrading enzyme. In addition, in the follow-up study, isotope labeling method was used to study the metabolism of HES *in vivo*, and on this foundation, infrared spectroscopy and H-NMR were used to study whether hydroxy groups in HES molecules react with carbonyl groups in heparin sulfate, hyaluronic acid, and heparin sulfate, and then to study the protective effect of HES on endothelial glycocalyx at the molecular level.

## Data Availability Statement

All datasets generated for this study are included in the article/**Supplementary Material**

## Ethics Statement

The animal study was reviewed and approved by This study was approved by Laboratory Animal Welfare and Ethics Committee of Third Military Medical University, and conformed to the Guide for the Care and Use of Laboratory Animal, published by the US National Institutes of Health (NIH Publication, 8th edition, 2011).

## Author Contributions

LL and TL contributed to conception, design, and revision of the article. YZ, JZ, YW, XX, and ZZ contributed to animal experiments. HZ contributed to animal experiments, sample collection and processing, and drafting and revision of the article. All authors read and approved the final article.

## Conflict of Interest

The authors declare that the research was conducted in the absence of any commercial or financial relationships that could be construed as a potential conflict of interest.
