# Supplementary material for: The Beneficial Effect of HES on Vascular Permeability and Its Relationship With Endothelial Glycocalyx and Intercellular Junction After Hemorrhagic Shock
Source: Front Pharmacol. 2020 May 8;11:597. doi: 10.3389/fphar.2020.00597 (PMC7227604; doi:10.3389/fphar.2020.00597)
Supplement: Supplementary file 1 [file Table_1.pdf]

	N	shock	LR	HES
	mean±SD	mean±SD	mean±SD	mean±SD
Weight (g)	208.89±6.47	210.81±6.08	209.48±5.58	209.55±5.74
Blood loss (ml)	/	6.18±0.25	5.95±0.25	6.02±0.28
Transfusion volume (ml)	/	/	11.9±0.49	12.0±0.56
Initial MAP (mmHg)	118.30±3.26	117.88±3.73	118.44±3.18	118.05±3.39
MAP after infusion (mmHg)	/	/	69.39±2.63	76.78±2.85
