# Supplementary figures and images for: The Beneficial Effect of HES on Vascular Permeability and Its Relationship With Endothelial Glycocalyx and Intercellular Junction After Hemorrhagic Shock
Source: Front Pharmacol. 2020 May 8;11:597. doi: 10.3389/fphar.2020.00597 (PMC7227604; doi:10.3389/fphar.2020.00597)

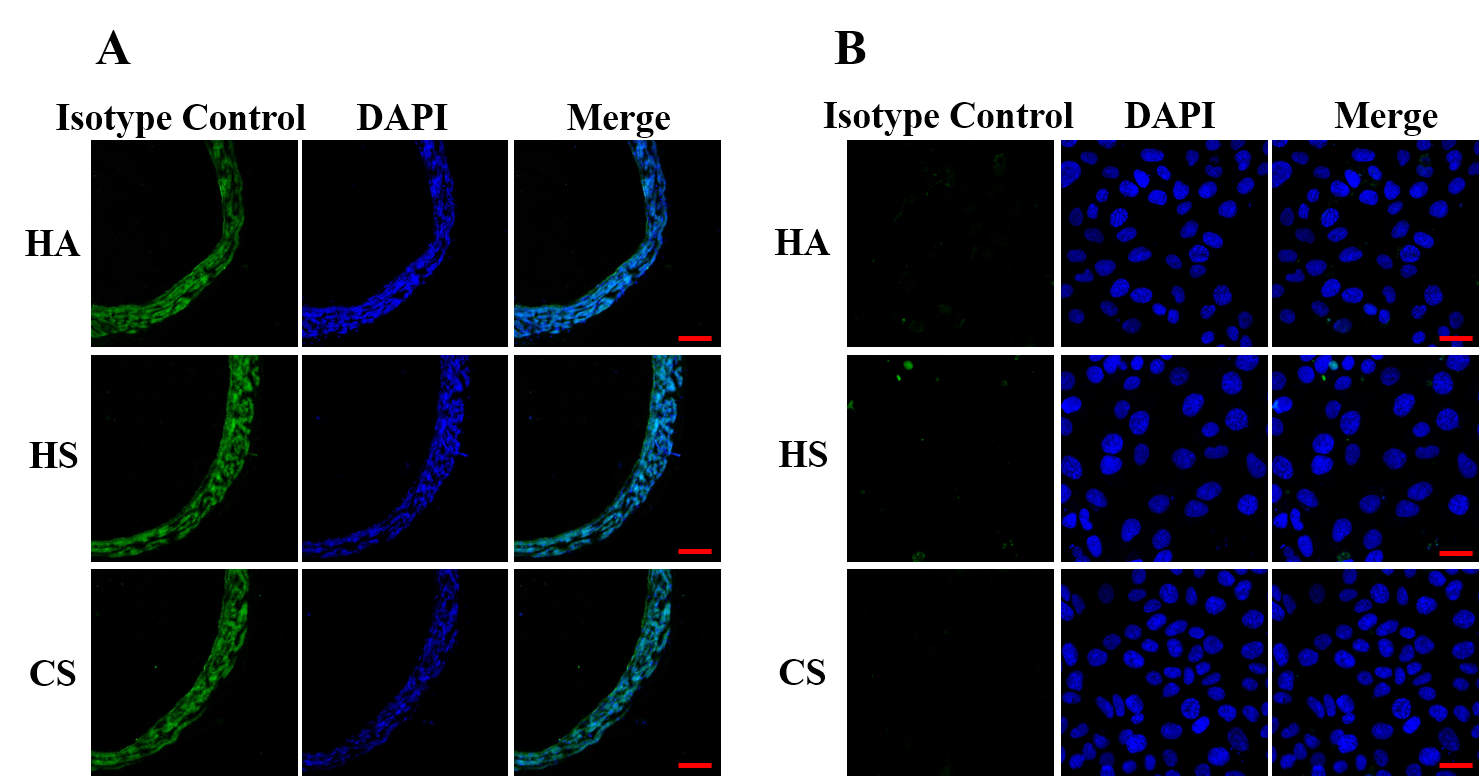

Supplement: Supplementary file 2 [file Image_1.tif]
